# Pituitary Neuroendocrine Tumors Extending Primarily Below the Sella and into the Clivus: A Distinct Growth Pattern with Specific Challenges

**DOI:** 10.3390/curroncol33010036

**Published:** 2026-01-08

**Authors:** Lennart W. Sannwald, Nina Kreße, Nadja Grübel, Andreas Knoll, Johannes Roßkopf, Michal Hlavac, Christian R. Wirtz, Andrej Pala

**Affiliations:** 1 Department of Neurosurgery, Ulm University, 89081 Ulm, Germany; nina.kresse@bkh-guenzburg.de (N.K.); nadja.gruebel@bkh-guenzburg.de (N.G.); andreas.knoll@bkh-guenzburg.de (A.K.); michal.hlavac@uni-ulm.de (M.H.); rainer.wirtz@uni-ulm.de (C.R.W.); andrej.pala@uni-ulm.de (A.P.); 2 Department of Neuroradiology, Ulm University, 89081 Ulm, Germany; johannes.rosskopf@bkh-guenzburg.de

**Keywords:** pituitary neuroendocrine tumors, transsphenoidal surgery, endoscopic endonasal surgery, clivus, sphenoid sinus, cavernous sinus, pituitary insufficiency, diaphragma sellae

## Abstract

Evaluation of pituitary neuroendocrine tumors remains complex depending on the exact growth pattern, involvement of critical neurovascular structures, pituitary function and endocrinological activity of the tumor. A predominant growth into the sphenoid sinus and clivus poses specific challenges. We reviewed 557 surgeries for pituitary neuroendocrine tumors in an endonasal endoscopic technique performed between 1 January 2015 and 31 August 2025 to identify 13 cases (2.3%). Clinical, radiological and surgical data were selected by chart review. Thirteen cases aged from 31 to 68 years with almost exclusively non-functioning or clinically silent tumors (92%) were identified. Clival infiltration was restricted to the dorsum sellae in 2/13 (15%), spread to the floor of the sphenoid in 6/13 (46%) and extended inferior to the sphenoid in 5/13 (38%) cases with a high rate of cavernous sinus (62%) and sphenoid sinus infiltration (69%). Complete resection was achieved in 31%, and the residual tumor was clival/sphenoidal in 5/13 cases or within the cavernous sinus in 6/13 cases. The diaphragma sellae was reported to be intact in 92% of cases, and postoperative transient arginine vasopressin deficiency did not occur. Pituitary neuroendocrine tumors predominantly growing below the sella and infiltrating the clivus and sphenoid present specific challenges with a high rate of preoperative pituitary insufficiency, frequent cavernous sinus infiltration and postoperative tumor residuals in the cavernous sinus, sphenoid bone and clivus which are sometimes difficult to delineate. The surgical approach must be tailored specifically to treat the clival infiltration zone to reduce the risk of recurrence.

## Simple Summary

Pituitary neuroendocrine tumors extending primarily below the sella into the clivus and sphenoid sinus are rare. Their specific challenges have largely been neglected in the literature. As one body of cavernous cancellous bone, the clivus offers a locus minoris resistentiae for diffuse tumor spread away from the classically addressed suprasellar region and optic chiasm. This study defines growth of these tumors in terms of three clival anatomical regions with different surgical accessibility and reports a high rate of preoperative pituitary dysfunction and infiltration of the cavernous sinus as well as the sphenoid sinus, posing a challenge to complete surgical resection with unexpected tumor residuals. Mechanisms of this growth pattern are discussed and help to plan surgical resection of these tumors.

## 1. Introduction

Pituitary neuroendocrine tumors are among the most frequent benign intracranial tumors and are generally amenable for surgery. Nevertheless, they pose specific challenges due to the complex interaction of endocrinological, ophthalmological and neurological sequelae of disease and therapy which are dictated by the anatomy of the individual pathology. Embedded in the sella turcica, growth of pituitary tumors is limited by bony walls anteriorly, inferiorly and posteriorly including additional double-layered dural coverage. While only a single-layered dural wall prohibits lateral growth, superiorly directed growth depends on individual variability in competency of the diaphragma sellae [1]. Historically, ophthalmological deficits due to suprasellar tumor extension have been the main therapeutic target in addition to endocrinological disease, and suprasellar lesions led to development of superior transtuberculum and transplanum extensions of the transsphenoidal approach [2,3]. Presuming tumor growth to take the path of least resistance, it was recognized at the beginning of the 20th century that—although being covered by the double-layered diaphragma sellae—a superior growth trajectory is facilitated by the diaphragmatic foramen as locus minoris resistentiae [1,4]. This was reported to be closed around the stalk in only 41.9%, partially open (up to 3mm) in 37.6% and widely open (more than 3mm) in 20.5% in 788 autopsy cases [5].

One of the first to draw attention to all types of parasellar tumor extension was Geoffrey Jefferson: he attributed the growth pattern and associated typical clinical presentation to (a) the nature of the adenoma and its growth tendency, (b) the state of fixation of the optic chiasm as well as (c) the shape of the pituitary fossa and diaphragma [6]. Especially in the second half of the 20th century, focus shifted increasingly towards lateral parasellar extension due to the relatively weak one-layered medial wall of the cavernous sinus [1,7,8]. However, at times true cavernous sinus wall infiltration is difficult to differentiate from naturally occurring lateral extension of the pituitary gland on preoperative imaging [9]. Focal invasion or thickening of the medial cavernous sinus wall was demonstrated intraoperatively even in small-growth-hormone- or ACTH-secreting adenomas with a low Knosp grade posing challenges for endocrinological cure, whereas diffuse wall destruction was shown mostly in nonfunctioning neuroendocrine tumors with a high Knosp grade and large size [9,10,11].

Although Jefferson also reported a case of a 19-year-old male patient with an adenoma infiltrating the basiphenoid and clivus, this type of tumor extension has received little attention to date [6]. Pituitary neuroendocrine tumors demonstrate a propensity for dural invasion in general [12]. Additionally, gradual erosion of the bony sella with a persistent borderline of cortical bone has been one of their diagnostic characteristics almost since the introduction of X-ray examination [13]. However, once this cortical lamella is perforated by the tumor or constitutionally, the caverns of the cancellous clival and petrous bone offer another path of least resistance with access to large parts of the skull base, where differentiation between adenoma and cancellous bone may be challenging, thus complicating surgical therapy [14,15]. This retrospective study analyzes characteristics of clinical and radiological presentation as well as surgical therapy of pituitary neuroendocrine tumors extending primarily below the sella and into the clivus.

## 2. Methods

### 2.1. Data Collection

**Figure 1:**
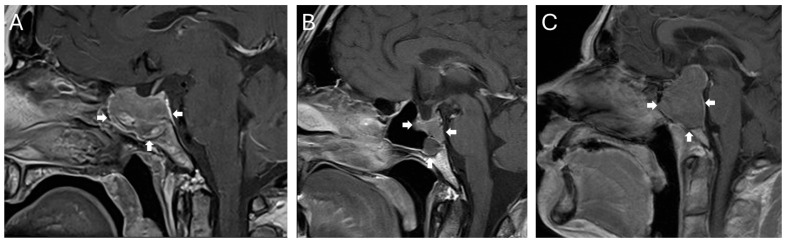
Examples of lesions included in this retrospective study. All lesions showed predominantly a growth from the sella into the clivus. While some tumors only spread from the sella inferiorly (**A**,**B**), others also demonstrated a minor growth into the suprasellar region (**C**). Infiltration of the sphenoid sinus is present in all three examples. The tumor extension is highlighted by white arrows.

A total of 557 surgeries for pituitary neuroendocrine tumors in an endonasal endoscopic transsphenoidal technique performed between 1 January 2015 and 31 August 2025 at the Department for Neurosurgery at Ulm University, Germany, were reviewed, and 13 cases (2.3%) were identified with predominantly sellar or infrasellar growth and extension into the clivus. Giant adenomas with diffuse infiltration into the clivus but also all other areas of tumor circumference were not included (for examples of tumors primarily extending below the sella, see Figure 1). Due to the often highly irregular and nodular suprasellar tumor growth, volumetry was not used to qualify the tumor as primarily extending below the sella and into the clivus. To qualify for an inclusion, more than 50% of the tumor mass was required to be within and below the sella, with only minor suprasellar nodules in relation to the tumor mass.

Consequently, age, sex, endocrinological and ophthalmological state and histology were collected by chart review.

Patients routinely received preoperative MR and high-resolution CT imaging to precisely define the individual skull base anatomy. Intraoperatively, the patients were positioned supine without head rotation and with rigid head fixation as intraoperative MRI is routinely performed to rule out unexpected tumor residual [16,17,18]. Transsphenoidal surgeries were performed in an endoscopic endonasal bi-nostril four-handed approach with neuronavigational guidance. Intraoperative electrophysiological monitoring of the abducens nerve was routinely applied in cases with clival infiltration. Perioperative substitution of hydrocortisone was only administered in patients with preoperative corticotrope insufficiency. Otherwise, cortisol and ACTH levels were checked on postoperative day 1 and 3 to assess the need for hydrocortisone substitution. Lumbar drains were not used in case of intraoperative leakage of cerebrospinal fluid after sellar reconstruction. Serum sodium was controlled daily throughout the hospital stay, and specific urine gravity was measured after every micturition to screen for arginine vasopressin deficiency and syndrome of inadequate ADH secretion.

**Figure 2:**
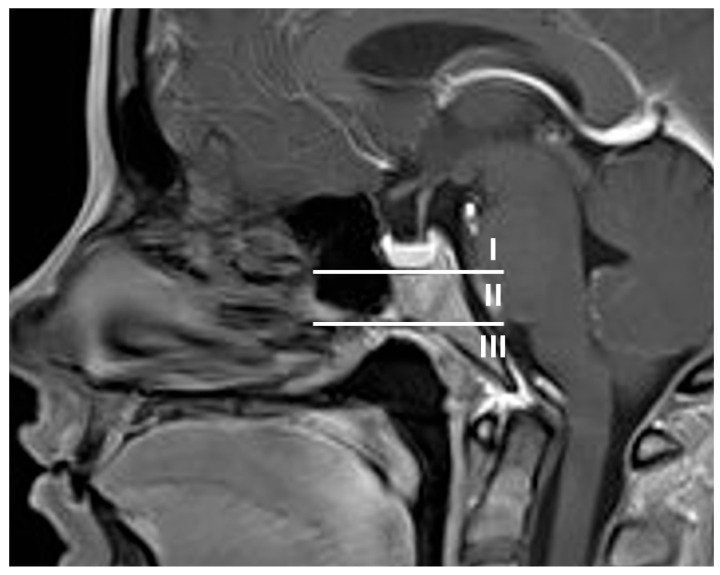
To assess surgical accessibility and facilitate surgical planning, the clivus was separated into three regions: region I: posterior clinoid to the floor of the bony sella, behind the pituitary; region II: from the sellar floor to the floor of the sphenoid sinus—there is a straight trajectory from the anterior wall of the sphenoid to this region; region III: inferior to the floor of the sphenoid sinus—this lower region proves particularly difficult to access surgically if only the sphenoid sinus is opened.

**Figure 3:**
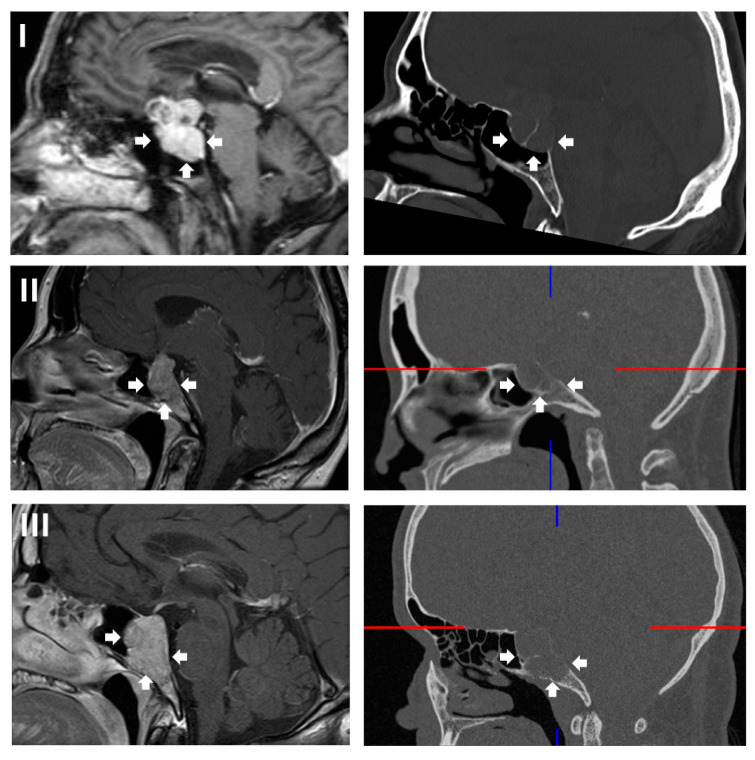
Examples of lesions within the respective clival regions as described in Figure 2. Left: contrast enhanced, T1-weighted cranial magnetic resonance imaging; right: bone window cranial computerized tomography. (**I**) The lesion is predominantly within the sella; the sellar bone and dorsum sellae are thinned diffusely; the tumor also presents smaller suprasellar extensions. Region II is spared by the tumor. (**II**) The lesion grows almost entirely in a downward direction within regions I and II. The sellar bone is largely intact, while the cortical lamella to the clivus is breached focally, enabling invasion of the cancellous clivus. The cortical lamella to the posterior fossa remains intact. (**III**) The tumor grows exclusively downwards and within all three clival regions. The anterior wall of the bony sella and floor of the sphenoid remain intact, while the sella floor is focally perforated, enabling tumor invasion of the cancellous clivus. A thin cortical lamella to the posterior fossa remains. The tumor extension is highlighted by white arrows. The color lines are an artefact of three dimensional image reconstruction.

**Figure 4:**
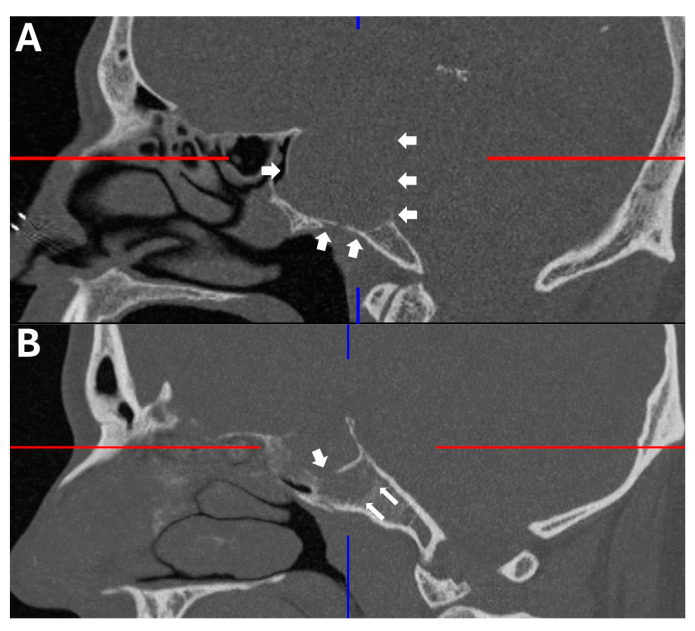
Representative example of the two types of clival infiltration: (**A**) The bony sella is diffusely eroded, enabling tumor invasion of the cancellous clivus. (**B**) The bony sella is focally perforated (three-dimensionally, the bone is focally perforated at multiple points), enabling tumor spread within the cancellous clivus and leaving cortical bone ‘within’ the tumor. Erosion of the cortical bone of the sella (**A**) and focal perforation (**B**) are highlighted by white arrows.

Preoperative and postoperative magnetic resonance and computerized tomography imaging were reviewed to assess tumor extension, specifically the type of clivus infiltration (focal or diffuse growth into the clivus, state of the cortical lamella), extent of resection, residual tumor and visibility of the pituitary gland. The type of sellar destruction was classified as diffuse only if almost the entirety of the sella floor was destroyed, while multifocal perforation of the sellar floor with consecutive spread into the cancellous clivus was classified as focal. In order to assess the extent of clival infiltration, three regions of tumor growth along the clivus were defined (Figure 2 and Figure 3): along the dorsum sellae from the posterior clinoid processes to the floor of the sella (region I), from the sellar floor to the floor of the sphenoid sinus (region II) and inferior to the floor of the sphenoid sinus (region III). Furthermore, the preoperative CT imaging was specifically reviewed for the type of clival infiltration (diffuse erosion and infiltration versus predominantly cancellous infiltration with intact cortical lamella of the sellar floor, Figure 4). Surgical notes (intactness of diaphragm, identification of pituitary gland and intraoperative cerebrospinal fluid leak) and all available follow-up data at last follow-up were reviewed as well. If a case was operated on between 2015 and 2025 due to a recurrence, the initial surgery was analyzed if data were available; otherwise, the recurrent case was analyzed. Thus, in two cases, an earlier operation was considered which was performed using a microsurgical transsphenoidal approach.

### 2.2. Statistical Analysis

Statistical analysis was restricted to descriptive statistics due to the small sample size. All research was carried out in accordance with the principles laid out in the Declaration of Helsinki. Scientific analysis and publication of cases from the registry were approved by the local ethics committee of Ulm University (identification number 137/16).

## 3. Results

### 3.1. Epidemiological Data

Thirteen cases were included in the analysis. The mean age at surgery was 56 years (31–74), and 8/13 patients were male. One of the 13 patients was a recurring tumor at initial surgery in our department. Follow-up ranged from 3 to 211 months (median 22 months). Preoperative anterior pituitary insufficiency was present in 8/13 patients (partial deficiency in 7 and total deficiency in 1), whereas 5/13 presented with preoperative visual deficits related to the tumor (Table 1).

Visual deficits showed a marked improvement postoperatively (only 2/13 patients with visual deficits at last follow-up). The high incidence of preoperative pituitary insufficiency, on the other hand, was aggravated by surgery, with 11/13 patients suffering from any form of pituitary insufficiency at last follow-up. Insufficiency at last follow-up was described by the treating endocrinologist for the gonadotrope axis in eight patients, for the somatotrope axis in one patient and for the thyreotrope and corticotrope axis in 10 patients, respectively.

Postoperatively, pathological examination revealed null cell adenoma in seven cases, one silent somatotrope adenoma, two silent corticotrope adenomas, two silent gonadotrope adenomas and one prolactinoma that developed pituitary apoplexy with near blindness (Table 2). In 3 out of the 13 cases, histological characteristics with a known association with invasiveness were present: a silent corticotroph adenoma and Ki-67 proliferation index up to 10% in two cases.

### 3.2. Growth Pattern

Ten out of the 13 patients presented with tumors that were predominantly sellar or infrasellar, while 3/13 patients suffered from macroadenomas with concentric sellar und suprasellar growth leading to clival erosion (Table 3). Two of those concentric macroadenomas were the only tumors growing into the upper clival region (posterior clinoid process to sellar floor, region I), whereas most of the lesions extended either into the middle clival region (level of the sellar floor to sphenoid floor, region II, 6/13) or the lower clival region (below the sphenoid floor, region III, 5/13). Of note, infiltration of the sphenoid sinus itself by the tumor was as common as any form of suprasellar extension (both 9/13) due to the specific growth pattern of this cohort.

Additionally, high-resolution CT imaging revealed that in 7/13 cases a focal perforation of the cortical lamella of the sellar bone at one or more areas was noted to facilitate clival growth, while this cortical lamella was intact in most areas despite diffuse tumor growth beyond this lamella along the cancellous bone. This presented as a partly intact cortical lamella within the tumor. In 6/13 cases, tumor growth led to diffuse erosion of most of the sellar floor. Furthermore, more than half of patients (8/13) presented with a high modified Knosp grade (3a, 3b or 4).

### 3.3. Surgical Results

Intraoperatively, a pituitary could be definitely identified in 7/13 cases compared to 6/13 on preoperative MRI. Moreover, the diaphragm remained intact throughout surgery in 12/13 cases, whereas five patients developed intraoperative leakage of cerebrospinal fluid (CSF), none of which demonstrated postoperative CSF leak. Postoperative electrolyte disturbance developed in one case only (transient syndrome of inadequate ADH secretion). Total tumor resection was only achieved in 4/13 cases. In 11/13 cases, intraoperative MRI was performed (2/13 received only postoperative MRI). Intraoperative MRI showed residuals in 9/11 cases (in two of these cases, resection of all residual in the cavernous sinus and sella was achieved after intraoperative MRI), whereas postoperative MRI showed residual tumor in 9/13 cases. After surgery, five cases demonstrated residual tumor in the clivus or sphenoid sinus, whereas six cases showed residual tumor in the cavernous sinus that was not resected intentionally to avoid increased morbidity. While the residuals in the cavernous sinus were purposefully not resected, the clival and sphenoidal residuals are principally resectable, highlighting the difficulty in differentiating infiltrated cancellous bone from healthy bone. Four of these five cases with clival/sphenoidal residuals were the only patients to develop significant tumor progression during follow-up. These four cases combined were operated on nine times, two received radiation (one radiosurgery, one fractionated radiotherapy) and were ultimately treated with temozolomide, underlining the challenge of containing these tumors. The time to tumor progression of these four out of five cases with clival/sphenoidal residuals was 10, 14, 102 and 105 months, respectively.

In summary, pituitary neuroendocrine tumors primarily infiltrating the clivus in our department comprised only 2.3% of cases and were almost exclusively clinically nonfunctioning adenomas (12/13) with common sphenoidal infiltration (9/13), a high rate of simultaneous cavernous sinus invasion (8/13) and a low rate of total resection (4/13). While tumor residual in the cavernous sinus was sometimes left deliberately, principally resectable clival or sphenoidal residual accounted for subtotal resection in 5/13 cases despite intraoperative MRI highlighting the challenge of identifying these tumors within cancellous bone both on imaging and intraoperatively. Furthermore, the clival and sphenoidal residuals in particular (4/5) proved prone to progression and necessitated repeated surgeries, radiation therapy and temozolomide in one case to contain tumor growth. There are two patterns of clival infiltration: focal perforation of the cortical lamella and diffuse growth within the cancellous bone of the clivus (7/13) and diffuse erosion of both cortical and cancellous bone (6/13). Furthermore, while concentric growth of macroadenomas (2/3) led to erosion of the clival region from the posterior clinoid process to the sellar floor (region I, 2/13), all other tumors also infiltrated the middle clival region (sellar floor to sphenoid floor, region II, 6/13) or the inferior clival region (inferior to sphenoid floor, region III, 5/13), which necessitates precise surgical planning for resection. Both perforation of the diaphragm (1/13 cases) and postoperative arginine vasopressin deficiency (0/13) were rare in this collective, in contrast to preoperative and postoperative pituitary insufficiency (8/13 and 11/13, respectively).

### 3.4. Illustrative Case

**Figure 5:**
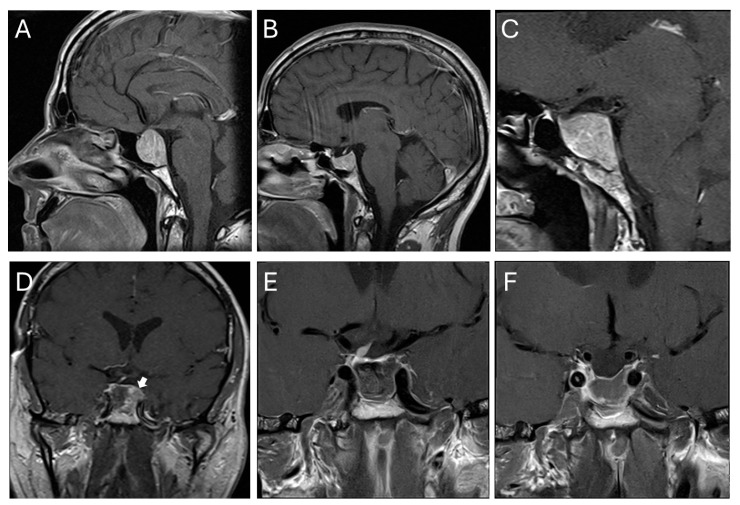
Illustrative case. (**A**) Preoperative imaging at the age of 48. The tumor predominantly grows within the sella and inferior to it with a minor suprasellar extension touching the optic chiasm. The tumor diffusely erodes the sellar bone, grows within regions I and II and respects the posterior fossa. (**B**) Early recurrence after three years. (**C**) Seventeen years after the first operation, the tumor demonstrated growth within all three clival regions. (**D**) Intraoperative MRI revealing residual tumor at the posterior part of the sella in contact with the left cavernous sinus. (**E**) Postoperative MRI after additional resection in the same surgical procedure. (**F**) MRI three months later. The white arrow in (**D**) shows tumor remnant on intraoperative MRI which was resected afterwards.

A 48-year-old male patient was diagnosed with a pituitary macroadenoma after a cranial MRI was performed due to a pituitary insufficiency (Figure 5A, patient number 13 in Table 1 and Table 3). The tumor expanded within the clival regions I and II and demonstrated a primary growth within the sella and clivus with a modified Knosp grade 2 lesion. Endocrinological work-up showed no evidence of hormone overproduction and total pituitary insufficiency. Due to the close contact to the optic chiasm (without visual deficits) and pituitary insufficiency, microscopic trans-sphenoidal resection was indicated and performed without complications. Histopathology revealed a null cell adenoma. The patient recovered without neurological deficit. The pituitary insufficiency however remained, and the patient received hydrocortisone, thyroxine and testosterone. Within the next three years, the patient showed an early recurrence (Figure 5B), however, without relevant suprasellar growth. Over the following 17 years, the tumor showed slow but steady growth (Figure 5C), now exclusively from the sella downwards into the clival regions I, II and III. Due to the constant growth and now significant size, a second surgery was performed via an endoscopic trans-sphenoidal approach. Intraoperatively, tumor delineation within the infiltrated cancellous bone proved difficult, particularly in the clival region III and towards the right cavernous sinus (Figure 5D). Intraoperative MRI helped to delineate residual tumor to be resected in the same surgical procedure (Figure 5E,F). Histopathology now revealed a silent gonadotrope adenoma. In both instances, the bony sella was diffusely eroded by the tumor, which invaded the cancellous clivus wherever it was in contact with the tumor and spread within it. Conversely, the tumor never showed any tendency to grow into the posterior fossa.

## 4. Discussion

This study of a small cohort with a specific and rare growth pattern of pituitary neuroendocrine tumors offers insights into their typical clinical and radiological presentation, their surgical challenges and develops a regional classification of the clival region to increase awareness of their pathoanatomy and determine the surgical approach. While lateral extension of pituitary neuroendocrine tumors into the cavernous sinus and suprasellar extension towards the optic chiasm have traditionally received much attention, clival infiltration is rarely specified [2,6,7,11,19].

Although extension of pituitary adenomas into the basisphenoid and clivus was first described in 1940 by Geoffrey Jefferson, it has gained attention only recently and is usually only characterized qualitatively as a measure of overall invasiveness of the adenoma as in the SIPAP-MR classification for pituitary adenomas [20]. However, access to the clival corridor offers unhindered potential growth within the cancellous bone up to the petrous apex and hypoglossal canal since the petroclival and spheno-occipital fissures are not composed of cortical bone [15]. This increases the extent of necessary surgical exposure significantly, and clival invasion was shown to be correlated with a higher rate of operative complications and recurrence [14]. Of note, differences in prevalence of clival infiltration of pituitary neuroendocrine tumors between this and other studies that report up to 10% derive from the specific growth pattern with predominant inferior extension into the clivus and sphenoid sinus reported in this study with exclusion of suprasellar giant adenomas also extending inferiorly [14,15].

While both clival and sphenoidal location as well as bone invasion have mainly been reported in the context of ectopic pituitary neuroendocrine tumors, there is no standardized system of describing an inferior growth pattern. Although the SIPAP classification includes infrasellar growth as a qualitative criterion, it does not comprise any further characterization of that growth to assess surgical accessibility [20]. In contrast, the three clival regions described in this study serve to evaluate the extent of clival growth, thus estimating the risk to the sometimes healthy pituitary (region I), the abducens nerve (transition from region I to II) and a potential invasion corridor into the petrous bone as well as the need to more extensively remove the bony floor of the sphenoid sinus (region III). This complements the well-established Knosp classification for lateral tumor extension. This classification offers a template that needs to be validated by future studies.

This study deliberately excludes giant adenomas that diffusely grow in all directions and focuses on tumors that predominantly grow below the sella and into the clival and sphenoidal region to investigate the clinical characteristics and surgical challenges of this area. Both diffusely spreading giant adenomas and ectopic adenomas within the clivus have been reported to be particularly aggressive and specific disease entities such as silent corticotroph adenomas characterized as prone to clival infiltration [21,22,23]. However, the observation that in many cases focal cortical bone perforations are combined with diffuse spread within cancellous bone implicates that it is not the invasiveness of the tumor but the unhindered expansion within the cancellous bone corridor that enables extensive growth in that region, while complete sellar destruction might follow secondarily. Intraoperatively, distinguishing healthy cancellous bone from infiltrated cancellous bone often proves challenging, leading to unexpected residuals in the clival region in 5/13 cases in this study, whereas residuals in the cavernous sinus are often accepted deliberately to reduce operative morbidity depending on the disease. Whether the observed propensity for recurrence with repeated surgeries, radiation therapy and even temozolomide therapy is sign of primary aggressiveness or secondary to repeated therapy remains to be determined in larger cohorts of this rare growth pattern. Nevertheless, it stands to reason that the surgical strategy should consist of systematic and region-oriented removal of the cancellous bone up to the dura or the next cortical bone layer after the tumor extension has been characterized according to three clival regions proposed on anatomical grounds: posterior clinoid process to sellar floor (superior region I, behind the pituitary), sellar floor to sphenoid floor (middle region II, straight surgical corridor through the sphenoid) and below the sphenoid floor (inferior region III, extended approach necessary).

Characterizing clival infiltration into the three suggested regions and observation of the type of perforation of sellar bone provides assistance in judging operative risks and planning the surgical approach. The importance of extension into region I (dorsum sellae) depends on the exact position of the pituitary: if the pituitary is pushed laterally, a trajectory for dissection past the pituitary is offered by the tumor growth. In the case of mostly infrasellar tumors without lateralized pituitary but infiltration into the dorsum sellae, dissection must proceed around the pituitary, which might be eased by sufficient exposure of region II (sellar floor to sphenoid floor) to dissect upward. However, it must be kept in mind that the transition zone between regions I and II at its most posterior aspect is the only area where the abducens nerve might be injured without transgressing laterally to the internal carotid artery at its entry into Dorello’s canal. Tumor extension into region III (below the sphenoid floor) necessitates more extensive drilling of the bony floor of the sphenoid sinus and dissection with an endoscope with angled optic, exposing the paraclival carotid artery. Additionally, this area offers an invasion corridor down to the petrous apex with proximity to the trigeminal nerve and petrous carotid. Differentiating infiltrated from unaffected cancellous bone remains an intraoperative challenge during dissection within the clivus. Radicality might be improved by drilling to the next unaffected cortical or dural border as predetermined on imaging. Particularly, the clival dura is usually neither breached nor infiltrated in contrast to the medial wall of the cavernous sinus, which is perforated by venous channels. In contrast, cortical bone might be perforated, highlighting the importance of preoperative high-resolution computed tomography to assess the involvement of dorsum sellae, clivus and carotid canal. Preoperative identification of the type of sellar bone perforation and remnants of cortical bone of the sella might serve as an intraoperative landmark for identification of a healthy pituitary. In the case of a focal perforation, the intrasellar tumor mass commonly displaces the pituitary away from the point of perforation and towards an intact area of the sellar floor. If a tumor residuum is to be expected, we tend to use a Hadad flap for skull base reconstruction in order to minimize the risk of delayed cerebrospinal fluid fistula after radiosurgery or fractionated radiotherapy. The stripped carotid artery should also be covered by vital tissue prior to radiosurgery in order to minimize the risk of rupture or pseudoaneurysm formation. All surgeries of this kind are routinely performed with neuronavigation, intraoperative doppler sonography and magnetic resonance imaging as well as electrophysiological monitoring of the abducens nerve.

The fact that the diaphragma was intraoperatively observed to be intact in 12/13 cases suggests that this growth pattern is the result of a lack of ‘natural decompression’ towards the suprasellar area as well as a better ability of sellar bone to remodel according to tumor growth in contrast to the bradytroph diaphragma sellae [1,4]. Consequently, it is hypothesized that the tumor (a) may exert excessive pressure on the pituitary, explaining the high rate of preoperative pituitary insufficiency, (b) slowly erodes or remodels sellar bone until a path of least resistance is created, which also (c) predisposes these lesions to break through the weakest point of the dural coverage around the sella, namely the medial wall of the cavernous sinus.

The high rate of pre- and postoperative pituitary insufficiency is noteworthy. Vascularization of the anterior pituitary lobe is considered to be provided mainly by the portal venous system of the superior hypophyseal artery along the stalk with venous perfusion pressure [24], whereas the posterior pituitary lobe is perfused by the inferior hypophyseal artery with arterial perfusion pressure via the meningohypophyseal trunk [25,26,27]. Only small parts of the anterior lobe are believed to be supplied with arterial perfusion pressure by small branches of the inferior hypophyseal arteries and McConnells inferior capsular arteries [27]. For this reason, section of the pituitary stalk leads to infarction of large parts of the anterior pituitary lobe [26,28]. However, with increasing tumor growth and intrasellar pressure, the portal venous system might be supplanted by an increasing arterial blood supply without hypothalamic releasing hormones from the capsular arteries and inferior hypophyseal artery. These vessels were demonstrated to play an increasing role in the vascularization of the sellar tumor angiographically [29], while dynamic MRI investigation showed replacement of the descending vascularization pattern with an ascending one in correlation with increasing tumor size [30]. Although speculative, this would (a) create a preoperative lack of hypothalamic releasing hormones and (b) put the remaining vascularization of the anterior pituitary lobe at high risk during tumor resection from an antero-inferior direction. Further study of preoperative tumor and pituitary vascularization with dynamic high-field magnetic resonance imaging or even angiography might enable a better correlation between perfusion pattern and endocrinological outcome.

However, both the role of diaphragmatic integrity and pituitary vascularization for endocrinological outcome and tumor growth pattern are speculative and remain to be elucidated by dedicated research.

Furthermore, intraoperative MRI may be a helpful tool to enhance the safe resection of adenomas invading the clivus. The clear delineation of anatomical borders, including internal carotid arteries, diaphragma and dura of the posterior fossa can be challenging due to the tumor’s invasiveness. Intraoperative MRI may improve the safety of additional resections, even in endoscopic procedures, similar to its application in the case of adenomas invading the cavernous sinus [31]. Since the extent of resection is associated with longer progression-free survival and in our serious predominantly clival residuals developed recurrence, it represents an additional technique that may improve outcomes [32]. Additionally, if tumor and resection reach the border between clival regions I and II, electrophysiological monitoring of the abducens nerve is advisable as has been practiced in the cavernous sinus compartment lateral to the internal carotid artery, since the abducens nerve is endangered both in the lateral compartment and towards the basilar venous plexus and Dorello’s canal [33,34].

While pituitary neuroendocrine tumors with clival, sphenoidal and cavernous infiltration demonstrate a propensity for invasion by definition, a high proliferation index was only demonstrated in 2 out of 13 cases. Thus, these tumors do not frequently fulfill criteria for the invasive and proliferative tumor with the highest recurrence rate as proposed by Trouillas et al. [35]. The complete surgical removal of the tumors presented in this study remains challenging. This in and of itself increases the likelihood of repeated surgeries and radiotherapy, leading to a gradual destruction of normal anatomy and a possibility of transformation into a more aggressive biology over time. The cause and effect of the recurrence rate and challenging treatment remain to be determined.

Limitations of this study pertain to the rare growth pattern, the associated small cohort size and the retrospective nature of the study. A longer follow-up will be necessary to indicate long-term differences in recurrence rates between clival and cavernous residuals as well as the potential for endocrinological recovery in this cohort.

## 5. Conclusions

In summary, pituitary neuroendocrine tumors with predominantly infrasellar, clival and sphenoidal growth present with a high rate of pituitary insufficiency and clinically silent adenomas. The clival extension is suggested to be categorized according to three clival regions to simplify surgical planning: posterior clinoid process to sellar floor (region I), sellar floor to floor of the sphenoid sinus (region II) and below the sphenoid sinus floor (region III). The high rate of intact diaphragm (12/13) suggests that the tumor grows inferiorly and laterally due to a lack of ‘natural decompression superiorly’. These tumors are regularly associated with cavernous sinus infiltration and a high Knosp grade (8/13). Once the tumors reached the cancellous bone of the clivus via focal or diffuse perforation of the sellar bone, a locus minoris resistentiae is established to enable tumor growth without affecting the posterior fossa dura. Delineation of the tumor within cancellous bone may prove challenging, leading to unexpected residuals (5/13). Preoperatively, specific definition of the type and extent of clival infiltration and the relation to the sixth nerve is necessary. An extensive resection of cancellous bone up to the next dural or cortical bone surface should be considered to ensure adequate resection. Intraoperative MRI and electrophysiological monitoring of the abducens nerve may make complete resection more feasible and safe.

## Figures and Tables

**Table 1:** Clinical presentation and outcome. Endocrinological outcomes are described more specifically in the text. Legend: M = male; F = female; temp defect = temporal field defect; bitemp defect = bitemporal field defect; age is given in years; Preop Pit Def = preoperative pituitary deficiency; Postop Pit Def = postoperative pituitary deficiency; Preop Vis Def = preoperative visual deficit; Postop Vis Def = postoperative visual deficit.

Pat	Age	Sex	Preop Pit Def	Postop Pit Def	Preop Vis Def	Postop Vis Def
1	50	F	No	Total	No	No
2	46	M	Partial	Total	No	No
3	31	M	Partial	No	No	No
4	50	F	Not tested	Partial	Near blind left	No
5	68	F	Partial	Total	Temp defect	Concentric defect
6	74	M	Partial	Total	Bitemp defect	Bitemp defect
7	71	M	Partial	Total	Bitemp defect	No
8	75	F	No	Partial	Not related	Not related
9	64	M	Partial	Total	Temp defect	No
10	47	F	Partial	Partial	No	No
11	58	M	No	No	No	No
12	52	M	No	Partial	No	No
13	48	M	Total	Total	No	No

**Table 2:** Tumor pathology.

Tumor Pathology	Number of Patients (n = 13)
Null cell adenoma	7
Silent somatotrope adenoma	1
Silent corticotrope adenoma	2
Silent gonadotrope adenoma	2
Prolactinoma	1

**Table 3:** Growth pattern and state of diaphragma sellae at intraoperative inspection.

Pat	Region of Clival Infiltration	Sellar Destruction	Diaphragma Intraop.	Mod. Knosp Grade	Sphenoid Sinus Infiltration
1	I–II	Focal	Intact	4	Yes
2	I–II	Focal	Intact	2	Yes
3	I–III	Focal	Intact	3b	Yes
4	I	Diffuse	Intact	4	Yes
5	I–II	Diffuse	Intact	3	Yes
6	I–II	Diffuse	Intact	4	Yes
7	I	Diffuse	Intact	2	No
8	I–III	Focal	Intact	3b	Yes
9	I–III	Diffuse	Intact	2	Yes
10	I–II	Focal	Perforated	1	No
11	I–III	Focal	Intact	3a	Yes
12	I–III	Focal	Intact	3a	No
13	I–II	Diffuse	Intact	2	No

## Data Availability

The data presented in this study are available on request from the corresponding author.
